# A novel, extreme low-cost poly (Erythrosine) modified pencil graphite electrode for determination of Adrenaline

**DOI:** 10.1038/s41598-023-31068-y

**Published:** 2023-03-20

**Authors:** S. D. Sukanya, B. E. Kumara Swamy, J. K. Shashikumara, S. C. Sharma, S. A. Hariprasad

**Affiliations:** 1 Department of P.G. Studies and Research in Analytical Chemistry, Alva’s College, Moodubidire, Dakshina Kannada, Karnataka 574227 India; 2 Department of P.G. Studies and Research in Industrial Chemistry, Kuvempu University, Jnana Sahyadri, Shankaraghatta, Shivmoga, Karnataka 577451 India; 3 National Assessment and Accreditation Council (Work Carried Out as Honorary Professor), Jain University, Bangalore, Karnataka 560 069 India; 4 Jain University, Bangalore, Karnataka 560 069 India

**Keywords:** Chemistry, Engineering, Physics

## Abstract

A simple, novel, and less cost yellow (Erythrosine) modified pencil graphite electrode (Po-ERY/MGPE) was successfully fabricated via electropolymerization method using cyclic voltammetric techniques. The fabricated Po-ERY/MGPE opted as a sensor for the detection of Adrenaline (ADR) in 0.2 M PBS (7.4 pH). This reported senor displayed excellent electrocatalytic activity, increased sensitivity, high stability, superior electron transfer kinetics in the oxidation of ADR once relative to BGPE. The significance of pH, scan rate, and impact of concentration was assessed at the sensor. As per the pH and scan rate study, redox routes carry the same number of electrons and protons, and electro-oxidation of ADR was adsorption controlled respectively. The LOD of ADR was found to be 0.499 µM. The DPV data indicate that there is a significant peak divergence among ADR and uric acid (UA) which could make it easier to determine them alone and simultaneously on the sensor. The described method has been employed for the determination of ADR in injection sample. Good recovery values indicate the efficacy and applicability of the sensor in detecting ADR.

## Introduction

In contemporary electro analytical chemistry, Graphite pencil electrodes (GPEs) are versatile analytical tools. GPE has gotten a lot of attention as a form of carbon electrode. GPEs has been employed in an expanding array of applications, primarily as sensors and biosensors, since the modern era, despite the fact that they were initially published in 1960^1,2^. It has good properties that are equivalent to glassy carbon and carbon paste electrode. GPE has extra advantages for the sensing of bioactive compounds by its lower costs, viability, mechanical robustness, high conductivity, renewability, easy disposability, low background current, wide potential range^3–8^.

Adrenaline (ADR) is a neurotransmitter, a hormone, and a prescription drug. ADR is a well-known catecholamine secreted by the endocrine system^9,10^. ADR content in blood is linked to a slew of physiological activities^11^. Aberrant ADR levels are linked to a various disease, including Alzheimer’s disease, Sclerosis, hypoglycemia, stress, thyroid hormone disorders, and Parkin’s disease^12–14^. It stiffens blood vessels, raising heart rate, dilates lungs, and is known to activate the sympathetic nervous system’s flight or fight mechanism^15,16^. ADR is a drug used to treat alleviate bronchiolitis, cardiac surgery, heart attack, heart blockage, hyper tension, asthma and anaphylaxis^17–19^. ADR and UA are biological substances that plays a vital function in human metabolism. They often reside in the central nervous system’s extracellular fluid and body fluids. Therefore, both in life science and in pathological research, the individual or simultaneous determination of ADR inexistence of UA is a crucial issue^20^.

To date, a lot of techniques have been used, including capillary electrophoresis^21^, fluorimetry^22,23^, liquid chromatography^24^, spectrophotometry^25^, chemiluminescence^26^ etc. These approaches are not only costly, and moreover time intensive and inconvenient. The electrochemical approach, on the other hand, has long been known as a simple, quick, low cost, high efficiency, and convenient method of analysis and it employs electrodes. The employ of bare electrodes, on the other hand, is reported to be plagued by low sensitivity and selectivity, making selective and simultaneous detection of compounds in a sample matrix problematic. The major solution to these problems related with bare electrodes has been electrode modification. Dyes have indeed been widely employed for electrode modification by electro polymerization method in this regard^27^.

**Figure 1:**
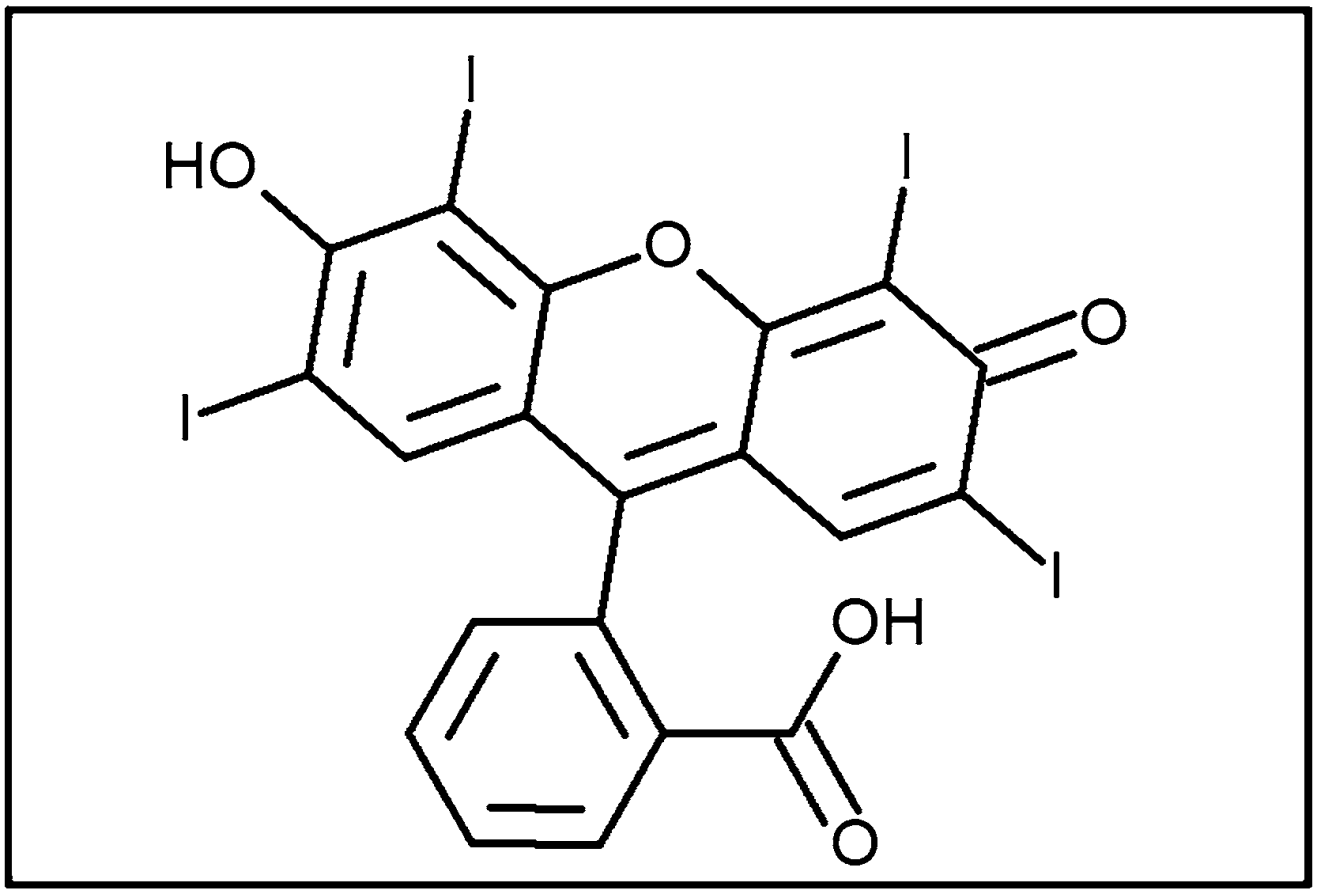
Structure of Erythrosine.

In this work, Erythrosine (ERY) (Fig. 1), a xanthene food dye was electropolymerized onto the GPE surface using the CV method^28^. The fabricated novel, simple and less cost Po-ERY/MGPE sensor plays a critical role in detecting ADR. Various parameters were analyzed, including pH effect, scan rate effect, and ADR concentration.

## Experimental part

### Chemicals and stock solutions

ADR and UA were provided by Himedia. ERY was procured from Sigma-Aldrich. Nice chemicals provided perchloric acid, NaH_2_PO_4_.H_2_O, NaOH, and Na_2_HPO_4_. The Camlin lead pencil rods with dimensions of 0.5 mm and 6 cm in length were purchased from the local book depot. The 25 × 10^–4^ M stock solutions of ERY, ADR, and UA were prepared in distilled water, 0.1 M perchloric acid, and 0.1 M NaOH respectively. A supporting electrolyte, 0.2 M PBS (7.4 pH) was prepared from Na_2_HPO_4_ and NAH_2_PO_4_·H_2_O. All the chemicals employed were analytical grade and were utilized without further refining.

### Instruments of investigation

All voltammetric (CVs and DPVs) assessments were obtained using CH instrument type 660 (CHI-660c model). The electrode setup consists of three electrodes: a working electrode (bare GPE or Po-ERY/MGPE), a counter electrode (Platinum), and a reference electrode (saturated calomel electrode). All voltammetric data were recorded at room temperature.

### Preparation of bare GPE

As bare GPE, lead pencil rod with a dimension of 0.5 mm and a length of 6 cm was employed. Copper wire was used to make the electrical connection at the pencil lead’s one end, and the surface was subsequently polished by rubbing it against weighing paper.

## Results and discussion

### Fabrication of Po-ERY/MGPE

**Figure 2:**
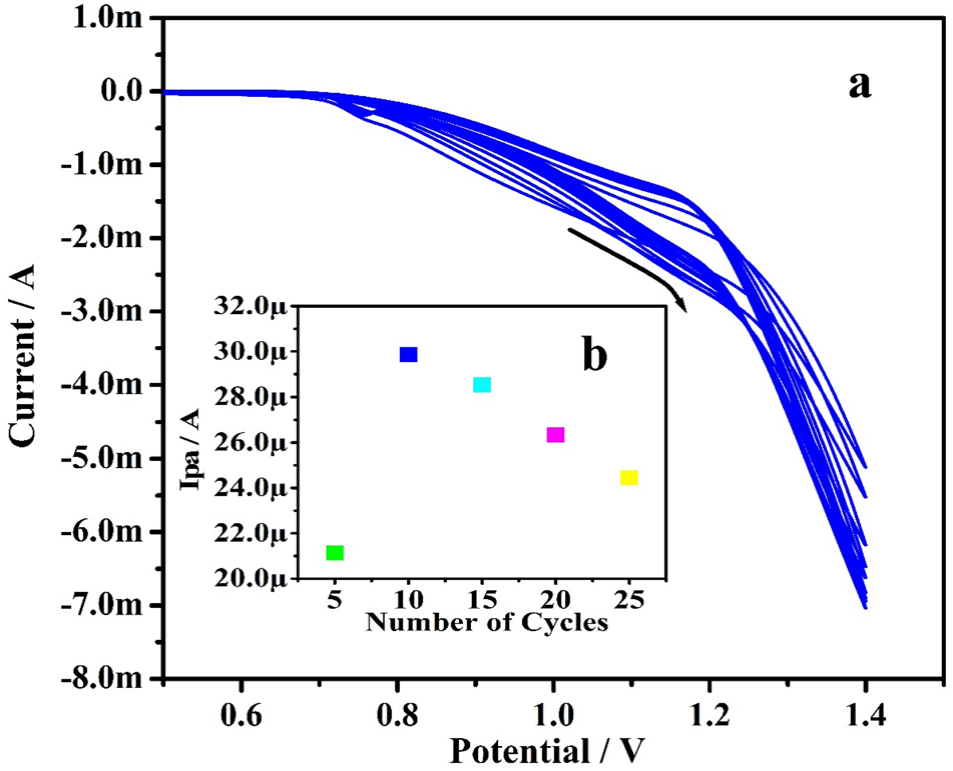
(**a**) CVs of fabricated Po-ERY/MGPE with NaOH (0.1 M) for 10 cycles at scan rate of 100 mVs^−1^. (**b**) Graph of Ipa Vs number of voltammetric scans.

The Po-ERY/MGPE was produced by electropolymerized a bare GPE (0.5 mm diameter) in 1 mM aqueous ERY prepared with 0.1 M NaOH (supporting electrolyte) in an electrochemical cell using the CV method. Figure 2a shows the results of CVs of the electropolymerization process at a potential gradient of − 0.6 to 1.4 V with a scan rate (ʋ) of 100 mVs^−1^ over 10 cycles. As the number of cycles increased, the voltammogram reduced and eventually became relatively steady, reflecting the accumulation of a uniform thin layer of ERY on the surface of bare GPE^29^. The thickness of the adherent film, which can be varied by adjusting the number of scan intervals during electropolymerization is strongly attributable to the modified electrode’s catalytic performance^30^. As indicated in the inset Fig. 2b, the maximal Ipa was attained at the 10 cycles, hence 10 cycles are being used as a standard for the electropolymerization on the bare GPE surface in all electrochemical analysis. The Eq. 1 was employed to compute an adequate estimated amount of surface coverage concentration or thickness of ERY on bare GPE surface, which was found to be 0.0124 M/cm^2^^27,31^.

1 $${\text{Ip = n}}^{2} {\text{F}}^{{2}} {\text{A}}\Gamma \upnu /4{\text{RT}}$$

where ‘n’ is number of electron transferred, ‘Ip’, ‘A’, ‘Г’, and ‘ʋ’ are peak current, area of the electrode, surface coverage area (M/cm^2^), and sweep speed respectively. F, R, and T are scientific terms.

### Electrochemical characterization of Po-ERY/MGPE

**Figure 3:**
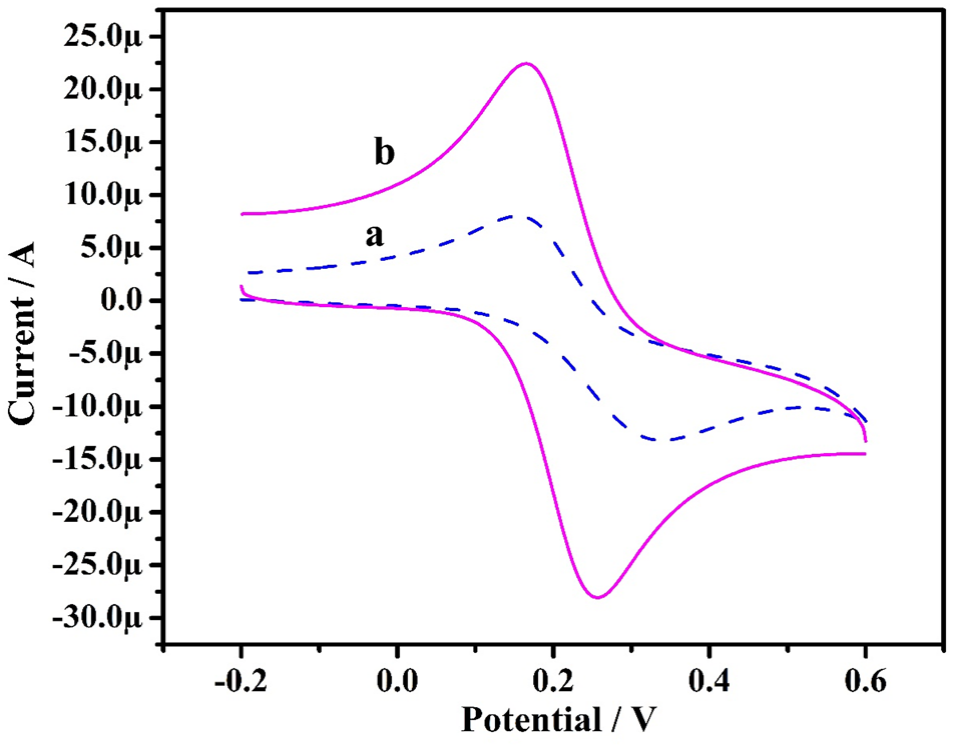
CVs of K_4_[Fe (CN)_6_] at bare GPE (curve ‘**a**’) and Po-ERY/MGPE (curve ‘**b**’) at a scan rate of 50 mVs^−1^.

The K_4_[Fe (CN)_6_] was employed as a redox probe in the CV technique to characterize the electrochemical performance of a produced electrode. The CVs for the electrochemical activity of K_4_[Fe (CN)_6_] at bare GPE (dashed line: curve ‘a’) and Po-ERY/MGPE (solid line curve ‘b’) in 1 M KCl as a supporting electrolyte with a scan rate of 50 mVs^−1^ was displayed in Fig. 3. As depicted in Fig. 3, bare GPE has a modest current response, whereas Po-ERY/MGPE has a higher maximum peak current than bare GPE. This finding suggested that the fabricated Po-ERY/MGPE had a larger electroactive surface area and create drastic increase in the rate of electron transfer. The electroactive surface area of bare GPE (0.022 cm^2^) and Po-ERY/MGPE (0.053 cm^2^) was estimated using Randles-Sevick’s Eq. (2). Which indicate that Erythrosine acts as an effective modifier contributing a larger surface area and promote the electron transfer between the electrode and solution.

2 $${\text{Ip }} = \, \left( {{2}.{69 } \times { 1}0^{{5}} } \right){\text{ n}}^{{{3}/{2}}} {\text{A D}}^{{{1}/{2}}}\upnu ^{{{1}/{2}}} {\text{C}}$$

where Ip is the peak current, n is the number of exchanged electrons, A is electroactive area in cm^2^, D is diffusion coefficient in cm^2^ s^−1^, ʋ is scan rate in Vs^−1^, and C is concentration of the electroactive species in mol cm^−3^.

### Electrocatalytic behavior of ADR at Po-ERY/MGPE

**Figure 4:**
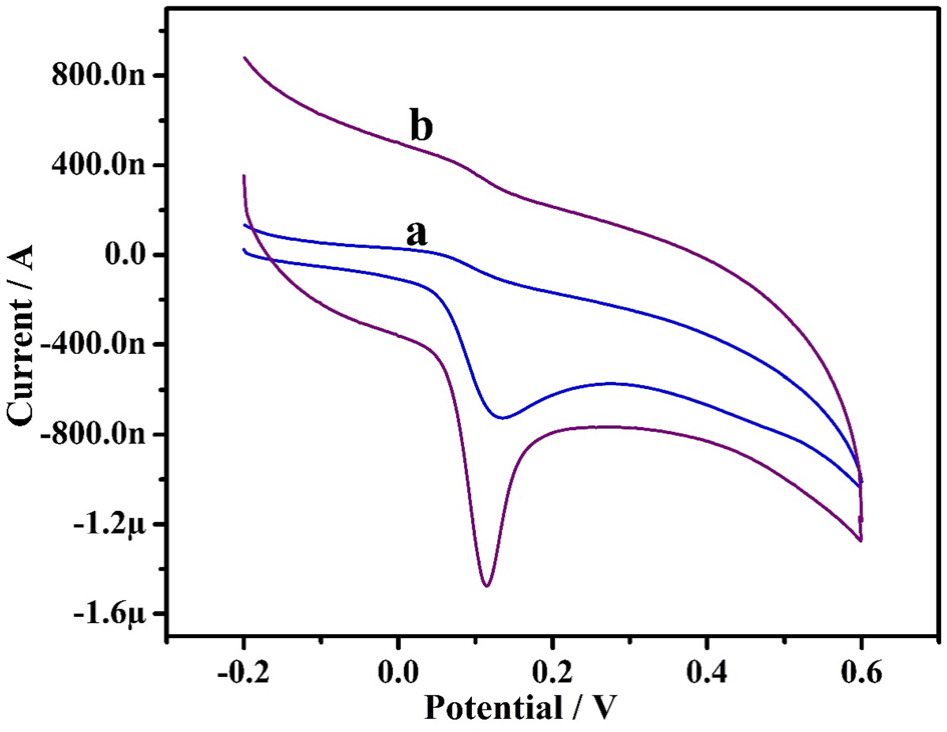
CVs of ADR in 0.2 M PBS (7.4 pH) at bare GPE (curve ‘**a**’) and Po-ERY/MGPE (curve ‘**b**’) at sweep rate of 50 mVs^−1^.

**Figure 5:**
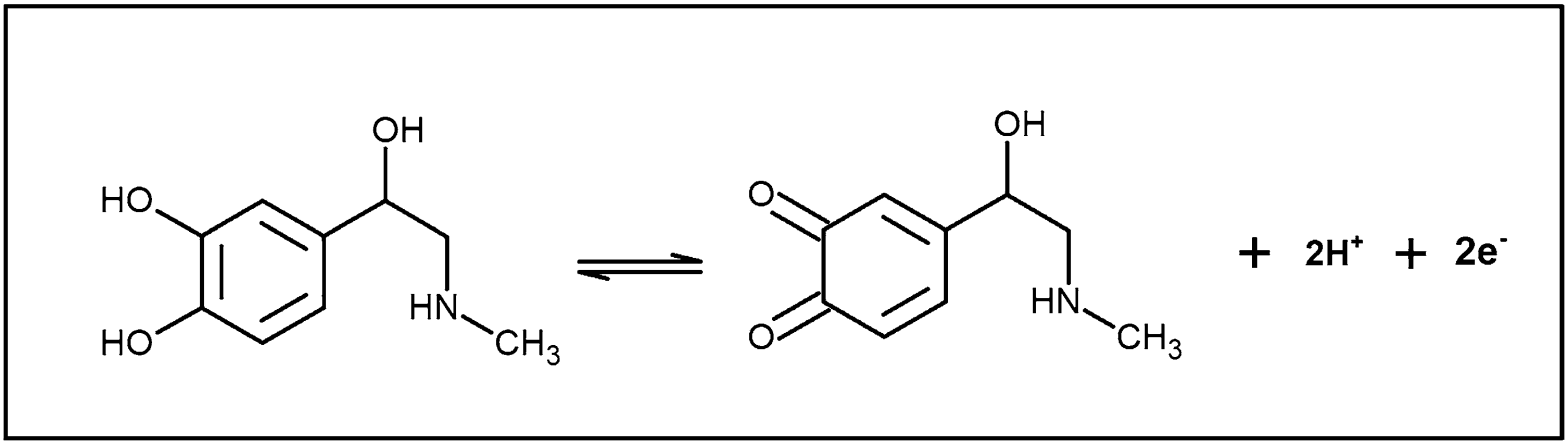
Oxidation of Adrenaline.

The electrochemical performance of 10 µM ADR was explored using CV technique at the bare GPE (curve ‘a’) and Po-ERY/MGPE (curve ‘b’) in 0.2 M PBS (7.4 pH) at potential ranging from − 0.2 to 0.6 V with scan rate of 50 mVs^−1^ shown in Fig. 4. ADR voltammetry responses depict broad massive oxidation waves at about 136 mV upon that at bare GPE, implying a slow and poor electron transport kinetics due to the fouling of electrode surface by oxidation reaction. Conversely, as contrasted to bare GPE, the projected PO-ERY/MGPE revealed a strong peak at 113 mV that is displayed favorably by 23 mV. These findings show that the ADR electron transport kinetics are speedier at Po-ERY/MGPE^10,32^. According to the established method, when the potential was initially swept from − 0.2 V to 0.6 V, a clear Ipa occur at 1.48 µA, and the electro behavior of ADR was completely irreversible, with no peak seen in the reverse sweep. The oxidation mechanism is attributed to two- electron oxidation of the hydroxy group, as detailed in Fig. 5.

### Significance of pH

**Figure 6:**
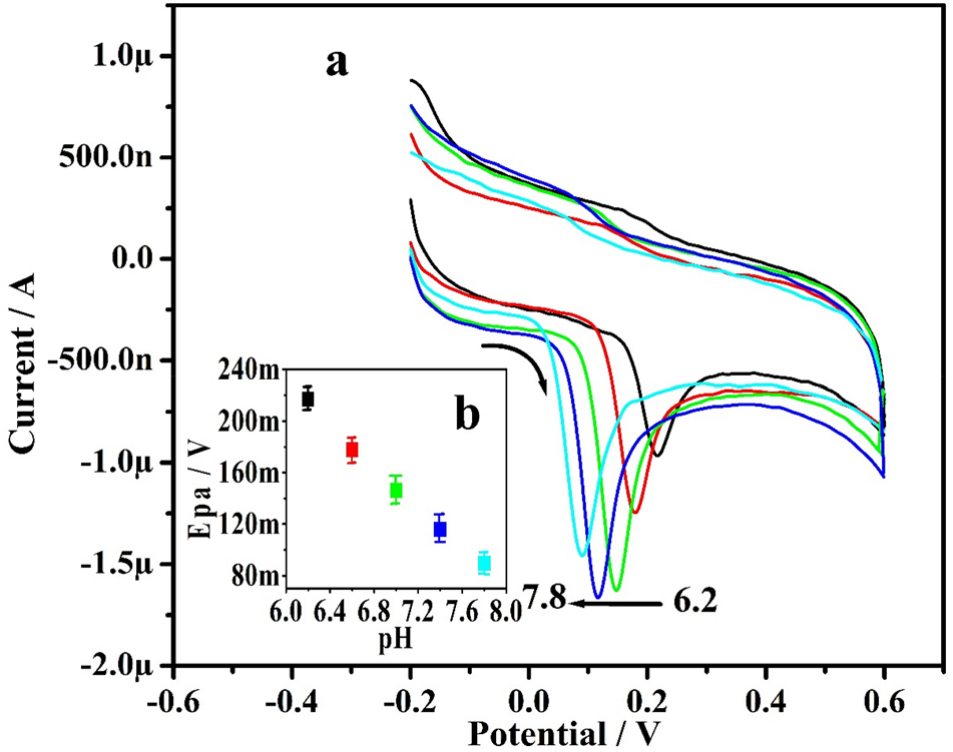
(**a**) CVs for ADR with pH series at Po-ERY/MGPE. (**b**) Graph of Epa Vs altered pH for ADR.

The contribution of supporting electrolytes in the development of sensors is critical for the analytes. At modified electrodes, pH has been shown to have a considerable influence on electron transport performance. As a result, it is often crucial to investigate the effect of buffer solution pH on analyte perception at modified electrode. Figure 6a depict the achieved CVs for 10.0 µM ADR in 0.2 M PBS of pH-adjusted from 6.2 to 7.8. As the pH increased, the oxidation peak potential of ADR switched to a more negative potential with difference in peak current. The graph of Epa for ADR Vs pH (inset Fig. 6b) revealed a linear connection with regression equation Epa (mVs^−1^) = 0.703–0.058 (pH), (R^2^ = 0.997) and slope of 0.058 V/pH, confirming that the oxidation of ADR comprises the same number of electrons and protons^20,33^.

### Impact of scan rate

**Figure 7:**
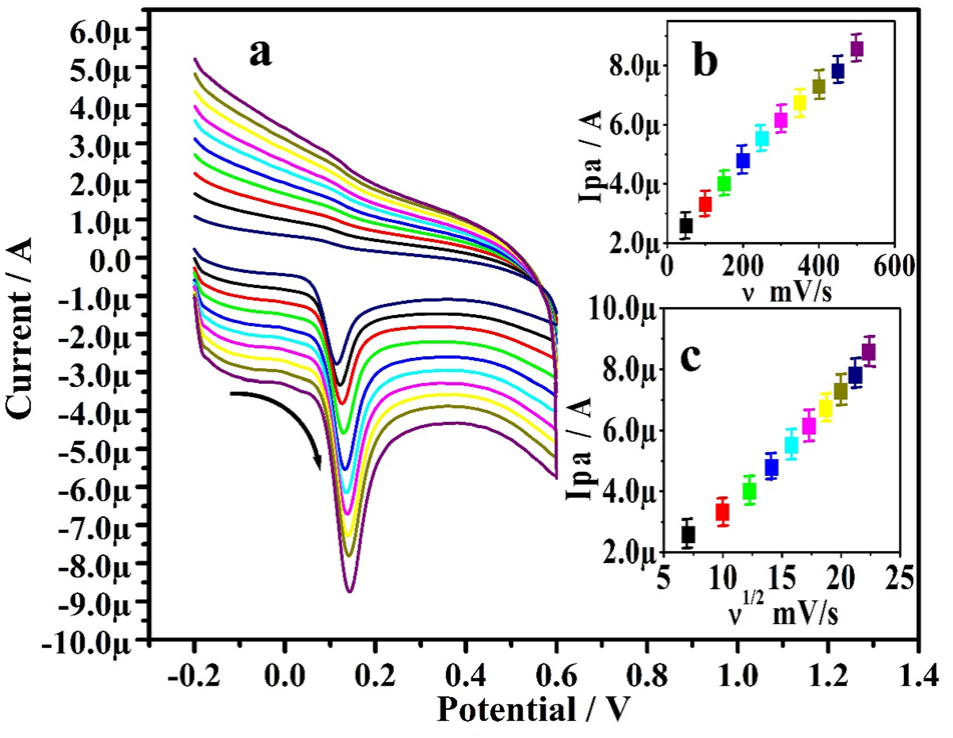
(**a**) CVs documented for ADR (10 µM) in 0.2 M PBS (7.4 pH) at Po-ERY/MGPE with various scan rate (50–500 mVs^−1^). (**b**) Graph of Ipa Vs scan rate of ADR (10 µM) in 0.2 M PBS (7.4pH). (**c**) Graph of Ipa Vs square root of scan rate of ADR (10 µM) in 0.2 M PBS (7.4pH).

The influence of scan rate from 50 to 500mVs^−1^ on electrochemical analysis of 10 µM ADR in 0.2 M PBS (7.4 pH) was explored using the CV technique, as seen in Fig. 7a. It was observed that as the scan rate increases the corresponding oxidation peak currents also increased with slight shift in peak potentials in accordance with Randles–Sevcik's relationship. Adsorption at Po-ERY/MGPE for ADR controls the electrode phenomenon, as evidence by the good linearity of the Ipa Vs scan rate (inset Fig. 7b) and Ipa Vs square root of scan rate (inset Fig. 7c) regression equations Ipa (µA) = 13.11 ʋ (Vs^−1^) + 2.06 (R^2^ = 0.998) and Ipa (µA) = 39.48 ʋ^1/2^ (Vs^1^) − 5.96 (R^2^ = 0.994)^34^.

### Concentration study of ADR

**Figure 8:**
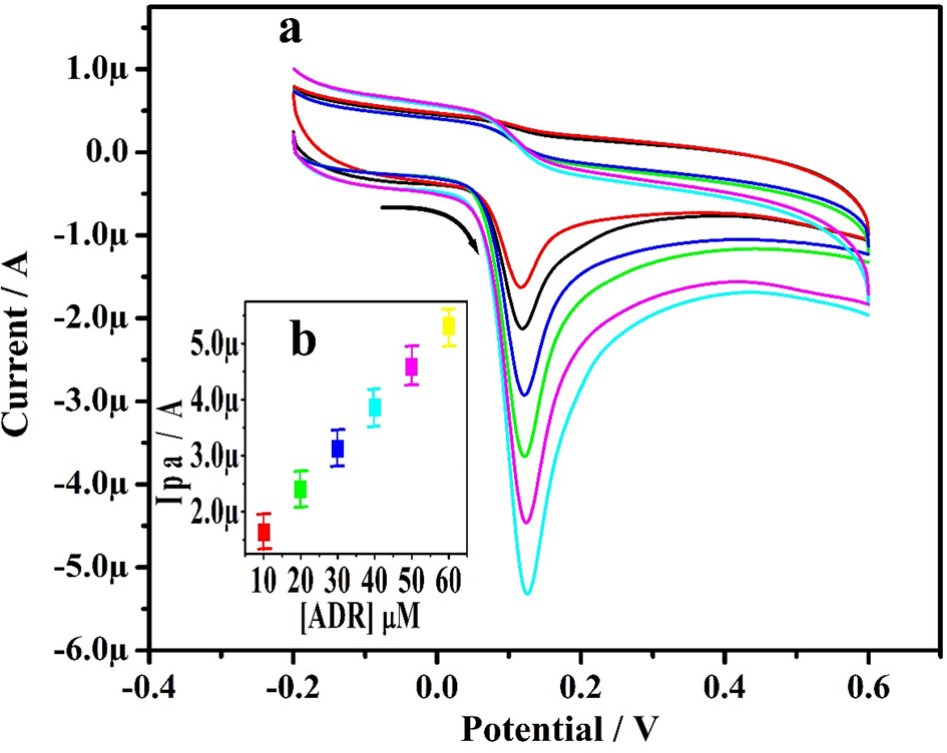
(**a**) CVs documented for different concentration of ADR (10–60 µM) using 0.2 M PBS (7.4 pH) at Po-ERY/MGPE. (**b**) Graph of Ipa Vs ADR concentrations.

Figure 8a presents CVs of ADR in 0.2 M PBS (7.4 pH) at 50 mVs^−1^ with a progressive increase in ADR levels from 10 to 60 µM achieved at Po-ERY/MGPE. Ipa of ADR increases as the ADR levels rise (inset Fig. 8b). With the regression equation of Ipa (µA) = 0.073 (µM) + 0.91(R^2^ = 0.999), it offers better linearity. Equations (3) and (4) were utilized to define the LOD and LOQ^35^. According to the estimation, the LOD is 0.499 µM and LOQ is 1.66 µM. Table 1 compares the LOD of Po-ERY/MGPE for ADR with that of other published sensors^14,20,34,36–41^. When we compare our findings to the literature, we find that our current study yielded better LOD results.

3 $${\text{LOD }} = {\text{ 3S}}/{\text{M}}$$

4 $${\text{LOQ }} = { 1}0{\text{S}}/{\text{M}}$$

**Table 1:** LOD of the proposed electrode in comparison to earlier ADR sensors.

Sl. no.	Electrode	LOD in µM	Method	References
1	Poly (safranin) modified carbon paste electrode (PSAF-MCPE)	0.61	CV	^14^
2	2-Hydroxybenzimidazole modified carbon paste electrode	3.0	CV	^20^
3	Poly(vanillin) modified carbon paste electrode	5.4	CV	^34^
4	Gold nanoporous film modified gold electrode	19	CV	^36^
5	Gold nanostructures on self assembled monolayer (Au/SAMs/AuNRs)	4.5	CV	^37^
6	Nanoporous spongelike Au–Ag electrode	5.05	CV	^38^
7	Nanoporous thin Au films	2.42	DPV	^39^
8	Carbon nanotube modified carbon film electrodes	0.9	DPV	^40^
9	Carbon paste electrode modified with iron phthalocyanine	0.5	DPV	^41^
10	Poly (Erythrosine) modified pencil graphite electrode (Po-ERY/MGPE)	0.499	CV	This work

### Simultaneous electro analysis of ADR and UA

**Figure 9:**
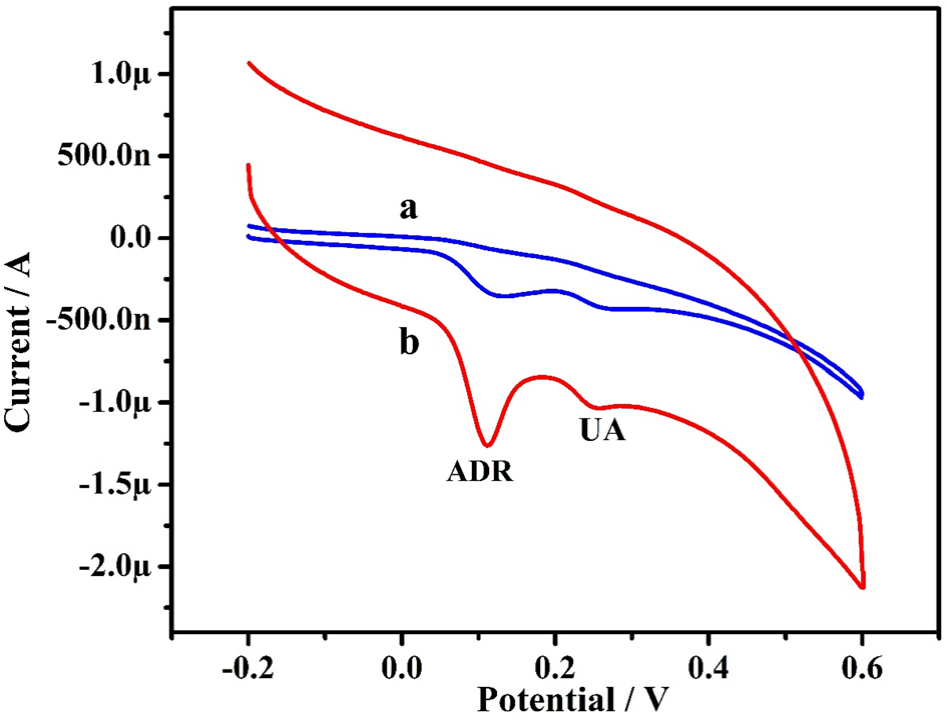
Resulted CVs for simultaneous studies of ADR (0.1 mM) and UA (0.1 mM) at bare GPE (curve ‘a’) and Po-ERY/MGPE (curve ‘b’).

The goal of this analysis was to utilize the developed electrode to assess ADR in the presence of UA in a selective and sensitive manner. The CVs obtained at sweep speed of 50 mVs^−1^ at bare GPE (curve ‘a’) and Po-ERY/MGPE (curve ‘b’) in 0.2 M PBS (7.4 pH) for a mixture of ADR (0.1 µM) and UA (0.1 µM) are illustrated in Fig. 9. At bare GPE, low current intensities with weak sensitivity and selectivity were measured. Po-ERY/MGPE also exhibited substantial current intensities with greater sensitivity and selectivity for ADR and UA oxidation at 0.111 V and 0.254 V, respectively, under the same circumstances. As a result, the built in Po-ERY/MGPE serves as an excellent ADR sensor.

### Interference study

**Figure 10:**
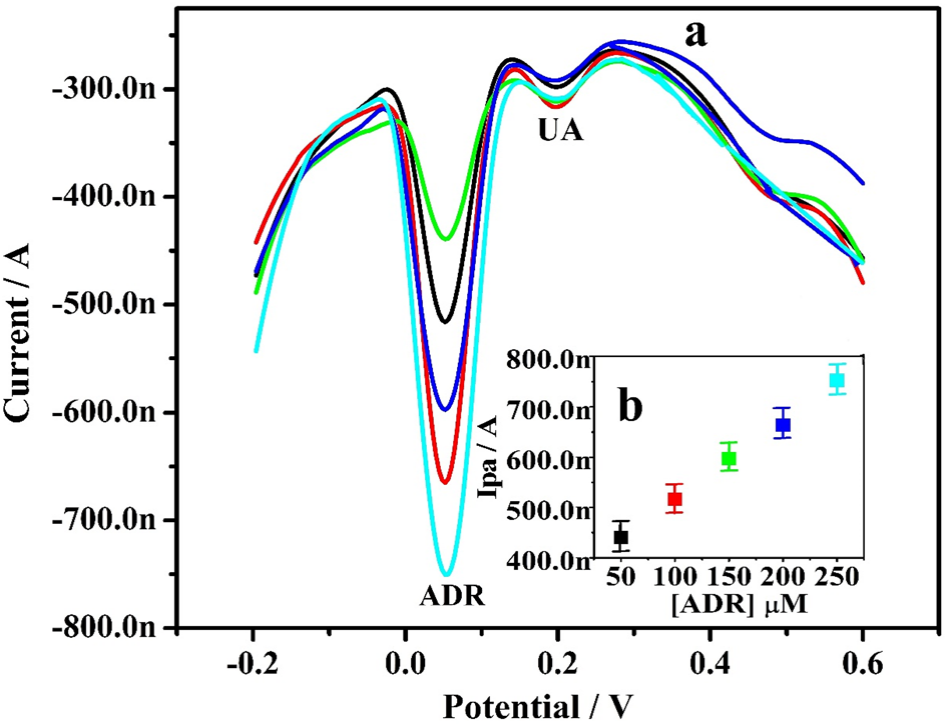
(**a**) DPVs of ADR at altered concentrations (50–250 µM) in 0.2 M PBS (7.4 pH) on Po-ERY/MGPE. (**b**) Plot of Ipa Vs concentration of ADR.

**Figure 11:**
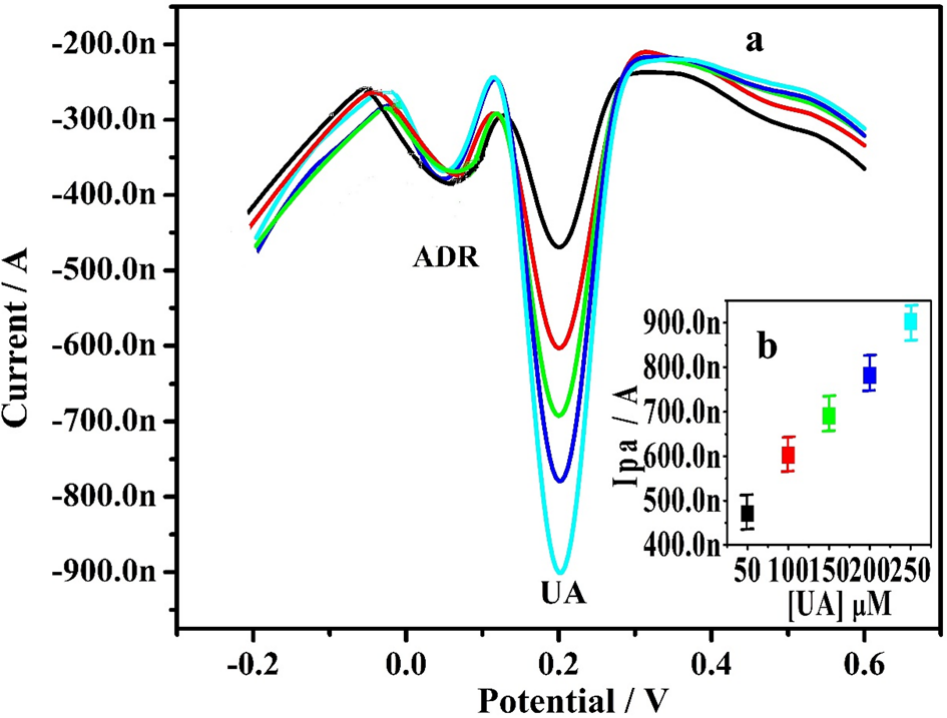
(**a**) DPVs of UA at altered concentrations (50–300 µM) in 0.2 M PBS (7.4 pH) on Po-ERY/MGPE. (**b**) Plot of Ipa vs concentration of UA.

The interference analysis was most significant in deciding the efficacy of the fabricated electrode and the DPV method was used for the study. The DPVs curve is depicted in Fig. 10a for a range of ADR concentrations (50–250 µM) while preserving UA (50 µM) constant. Likewise, the concentration of UA (50–300 µM) was varied while the ADR concentration remain constant (Fig. 11a). A linear plot of Ipa vs concentration of ADR (inset Fig. 10b) and Ipa vs concentration of UA (inset Fig. 11b), producing the regression equation Ipa (nA) = 0.073 (µM) + 9.14 (R^2^ = 0.999) and Ipa (nA) = 0.020 (µM) + 3.76 (R^2^ = 0.997), respectively. According to the above-mentioned experimental data, as the level of one analyte was increased, the Ipa climbed wonderfully, but the Ipa and Epa of constant analyte remained unchanged. Because the oxidation of ADR does not influence the variance of the other analytes, our data suggest that the accurate and precise estimation of ADR at Po-ERY/MGPE is achievable.

### Stability and reproducibility of Po-ERY/MGPE

To examine the sensor’s stability, the Po-ERY/MGPE was subjected to 10 successive cycles for 10 µM ADR in 0.2 M PBS with scan rate of 50 mVs^−1^ by CV method. The computed percentage of degradation indicates 93.72% of the primary peak current signal, indicating adequate stability. The reproducibility of the MGPE was examined by fabricating four separate MGPE under the same circumstances. The RSD value obtained to be 4.7% validates the good reproducibility.

### Real sample analysis

In addition to the aforementioned studies, the Po-ERY/MGPE sensing potential towards ADR in an injection sample was assessed using the standard addition method. The injection sample was procured from Harson Laboratories, which had a defined concentration of 1.8 mg/mL ADR and was used after a sufficient dilution in 0.1 M perchloric acid. Table 2 displays, 4 consecutive ADR concentrations in the range of 10 to 40 µM, result in a strong recovery in the range of 97.0 ± 0.982 to 99.9 ± 0.025 percent. These results demonstrate Po-ERY/MGPE’s sensing potential for ADR analysis in an injection sample.

**Table 2:** Result of recoveries of ADR at Po-ERY/MGPE in an injection sample.

Sample added (µM)	Found (µM)	Recovery (%)
10	9.7	97.0 ± 0.982
20	19.8	99.0 ± 0.398
30	29.6	98.6 ± 0.791
40	39.8	99.9 ± 0.025

## Conclusion

In conclusion, we proposed a simple and efficient way for fabricating of Po-ERY/MGPE. The core attractive features of Po-ERY/MGPE include the prompt modification process, increased sensitivity, and low detection limits. The developed sensor was used for specific and simultaneous detection of ADR and UA. According to DPV studies, highly distinct and defined peak separation between ADR and UA could be enabling both individual and simultaneous determination of each compound on the sensor. Po-ERY/MGPE exhibits good sensitivity, stability and reproducibility. In real sample analysis, the developed sensor recovered ADR with high accuracy. As a result, Po-ERY/MGPE will have a high opportunity in sensor field.

## Data Availability

All data generated or analysed during this study are included in this published article.
